# pH-Sensitive Dairy-Derived Hydrogels with a Prolonged Drug Release Profile for Cancer Treatment

**DOI:** 10.3390/ma14040749

**Published:** 2021-02-05

**Authors:** Oksana A. Mayorova, Ben C. N. Jolly, Roman A. Verkhovskii, Valentina O. Plastun, Olga A. Sindeeva, Timothy E. L. Douglas

**Affiliations:** 1 Institute of Nanostructures and Biosystems, Saratov State University, 83 Astrakhanskaya st., 410012 Saratov, Russia; r.a.verhovskiy@mail.ru (R.A.V.); voplastun@gmail.com (V.O.P.); o.sindeeva@skoltech.ru (O.A.S.); 2 Engineering Department, Lancaster University, Gillow Av., Lancaster LA1 4YW, UK; bcnjolly001@gmail.com; 3 Skolkovo Institute of Science and Technology, Skolkovo Innovation Center, Building 3, 143026 Moscow, Russia; 4 Materials Science Institute (MSI), Lancaster University, Gillow Av., Lancaster LA1 4YW, UK

**Keywords:** whey protein isolate, hydrogel, tannic acid, anticancer scaffold

## Abstract

A novel versatile biocompatible hydrogel of whey protein isolate (WPI) and two types of tannic acid (TAs) was prepared by crosslinking of WPI with TAs in a one-step method at high temperature for 30 min. WPI is one common protein-based preparation which is used for hydrogel formation. The obtained WPI-TA hydrogels were in disc form and retained their integrity after sterilization by autoclaving. Two TA preparations of differing molecular weight and chemical structure were compared, namely a polygalloyl glucose-rich extract-ALSOK 02-and a polygalloyl quinic acid-rich extract-ALSOK 04. Hydrogel formation was observed for WPI solutions containing both preparations. The swelling characteristics of hydrogels were investigated at room temperature at different pH values, namely 5, 7, and 9. The swelling ability of hydrogels was independent of the chemical structure of the added TAs. A trend of decrease of mass increase (MI) in hydrogels was observed with an increase in the TA/WPI ratio compared to the control WPI hydrogel without TA. This dependence (a MI decrease-TA/WPI ratio) was observed for hydrogels with different types of TA both in neutral and acidic conditions (pH 5.7). Under alkaline conditions (pH 9), negative values of swelling were observed for all hydrogels with a high content of TAs and were accompanied by a significant release of TAs from the hydrogel network. Our studies have shown that the release of TA from hydrogels containing ALSOK04 is higher than from hydrogels containing ALSOK 02. Moreover, the addition of TAs, which display a strong anti-cancer effect, increases the cytotoxicity of WPI-TAs hydrogels against the Hep-2 human laryngeal squamous carcinoma (Hep-2 cells) cell line. Thus, WPI-TA hydrogels with prolonged drug release properties and cytotoxicity effect can be used as anti-cancer scaffolds.

## 1. Introduction

Recently, much attention has been paid to hydrogels in drug delivery. In this regard, hydrogels must comply with principles such as biocompatibility, biodegradation, and non-toxicity. One common protein-based preparation used for hydrogel formation in the food industry is whey protein isolate (WPI), which we have recently begun to investigate as a hydrogel biomaterial for biomedical applications [1,2,3,4]. The major component of WPI is ß-lactoglobulin (approximate composition 74.1%) and the second major component is α-lactalbumin (23.0%) [5]. Whey proteins have been identified to have desirable properties because they consist of branched-chain amino acids which promote highly hydrated three-dimensional polymer networks in hydrogels [6]. Gelation occurs by increasing the temperature due to denaturation of native ß-lactoglobulin protein [7]. The process of whey protein aggregation consists of three stages, including conformational changes of the native protein structure, chemical reactions typically through disulfide bridges between intra- and interchain bonds and physical interactions like hydrophobic interactions, which leads to aggregation clustering and the formation of a spatial gel network [8]. The increased comparison of ß-lactoglobulin allows to fabricate more elastic WPI hydrogels with far superior mechanical properties compered to hydrogels based on whey protein concentrate. The important functional property of a WPI hydrogels is its high ability to retain water or body fluids within its structure. The WPI denaturing permits exposure of hydrophobic regions of the protein molecule, to which the hydrophobic regions of hydrophobic drugs can bind, resulting in increased drug solubility. Cytocompatible hydrogels have been successfully used to develop drug delivery systems due to their stimulus-sensitive response to external triggers, such as pH [9]. Hence, it would be desirable to combine the ability of WPI hydrogels to solubilize and carry hydrophobic drugs with pH responsiveness.

One class of hydrophobic molecules with biological activity are tannic acids (TAs). TAs are polyphenols closely related to our daily life: They are found in many fruits and vegetables consumed by humans and are used in the food industry and herbal medicine. Hydrolyzable tannins are one of three types of TAs that are formed by a carbohydrate (glucose, quinic acid, or other), in which OH-groups are partially or completely esterified with gallic acid or related compounds [10,11,12]. In this context hydrolyzable means that ester hydrolysis can occur, as opposed to acid-base hydrolysis (deprotonation). Hydrolyzable tannins can be extracted from various vegetable plants and trees. As a rule, TAs are considered non-toxic in small doses [13,14] and exhibit antitumor effects [15]. The presence of TA in natural components can reduce tumor necrosis factor levels [16] and weaken the inflammatory cytokine expression [17]. Previously, it was shown that TA crosslinked into a compacting collagen gel predominantly inhibited proliferation of high-melanoma A375 cells with metastatic potential [18]. In addition, ternary composite nanofibers containing tannic acid can be used as wound dressings in the case of recessive dystrophic epidermolysis bullosa, which often leads to the development of an aggressive form of squamous cell carcinoma [19]. TA has been shown to help crosslinking of gelatin and pectin derivatives due to the presence of a large number of hydroxyl groups in the polyphenol structure due to intermolecular H-bond formation, in which the polyphenols act as electron pair donors [20]. From the physicochemical point of view, polyphenols stabilize the secondary structure of proteins, increase their thermal stability and significantly reduce their biodegradability [21]. Recently, a comparative analysis was carried out of the ability of gellan gum hydrogels enhanced with polyphenols (including the ones investigated in our research, ALSOK 02 and ALSOK 04), to enzymatic mineralization and the hydroxyapatite formation [22]. TA inclusion inhibited the growth of human osteoblast-like Saos-2 cells on substrates of mineralized gellan gum hydrogel biomaterials with calcium phosphate and did not confer antibacterial activity against *Escherichia coli*.

In this study, we combined the beneficial properties of TAs and WPI to create new pH-sensitive cytocompatible hydrogels which display an anticancer affect. Two TAs of differing molecular weight and chemical structure (polygalloyl glucoses—ALSOK 02 and polygalloyl quinic acids—ALSOK 04) were compared using swelling tests at different pH values. We hypothesized that the addition of TAs would reduce the swelling of WPI hydrogels due to the aforementioned interactions between polyphenols and proteins. To our best knowledge, this combination of components has not yet been tested for biomaterial-related applications. We focused on the dependence of the swelling ability of hydrogels on pH of the medium, chemical structure, and concentration of TAs, which allowed a more prolonged release of TAs over several days. The behavior of hydrogels that are sensitive to external pH are especially in demand in the development of anticancer scaffolds. The cytotoxic activity of TA and WPI-based hydrogels were evaluated in vitro against the Hep-2 human laryngeal squamous carcinoma cell line (Hep-2 cells).

## 2. Materials and Methods

### 2.1. Materials

Phosphate buffered saline (PBS, 0.01 M), iron(III) chloride (tetrahydrate) were obtained from Sigma-Aldrich (Steinheim, Germany). AlamarBlue (Cell Viability Reagent) was obtained from Invitro-gen (Waltham, MS, USA). Hydrochloric acid and sodium hydroxide was purchased from Reakhim (Moscow, Russia) and used without further purification. WPI (BiPRO, Davisco Foods Int., Inc., Eden Prairie, MN, USA) with 97.7% protein and 75% BGL in dry matter (according to the specification) was used as described previously [6]. TAs (ALSOK 02; MW 1040 D; pentagalloyl glucose 20% by weight; ALSOK 04; MW 850 D) was purchased from Omnichem NV Belgium (Wetteren, Belgium). Millipore Milli Q water (18.2 MΩ cm^−1^) was used as an aqueous medium during all sets of experiments.

### 2.2. Methods

#### 2.2.1. Hydrogel Preparation

The hydrogel preparation was carried out by thermally-induced gelation. Hydrogels were fabricated with four different concentrations of TAs: 1.5, 3.0, 6.0, and 12.0 mg per mL which corresponds to the TA/WPI ratios were 0.0375/0.075/0.15/0.30 in the hydrogels; a control sample without the TA addition was also prepared. The required amount of TAs was added to the initial solution consisting of 40 mg per mL WPI [23]. All WPI hydrogels were prepared from a solution at pH 7.0. The protein–polyphenol solutions were left in the refrigerator overnight to remove excess air bubbles present. The solutions were transferred to plastic Petri dishes. Gelation was carried out at 90 °C for 30 min in an oven. Each cm^2^ of the Petri dish surface area was occupied by 0.31 mL hydrogel. The resulting hydrogels were then transferred to glass Petri dishes for further autoclaving at 121 °C for 15 min before any further characterization.

#### 2.2.2. Swelling Study in Phosphate Buffered Saline (PBS) with Different pH Values

The behavior of the swelling of the hydrogel samples was carried out in PBS at different pH values (pH 5, 7, 9). The desired basic and acidic pH values were obtained by pH adjustment using NaOH and HCl solutions, respectively. To measure the swelling, after autoclaving, samples of the excised hydrogel discs (diameter 3 mm) were dried at 80 °C for 1 h, then a dried sample with known weight was placed in 24-well plates and incubated in a solution (1:10). The swelling process took place at room temperature for up to 48 h. Swollen gels were periodically (1, 24, and 48 h) removed, blotted on dry filter paper to remove excess water and immediately weighed. Then, the mass increase (*MI*) was calculated as:

*MI* (%) = ((*Mt* − *Mo*)/*Mo*) × 100

where *Mt* is the weight of the hydrogel at a certain time, *Mo* is the initial hydrogel weight. All experiments were carried out with *n* = 6.

#### 2.2.3. Fourier Transform Infrared (FTIR) Spectroscopy

The chemical structure of the synthesized WPI hydrogels was investigated by using Fourier Transform Infrared spectroscopy using a Fourier-transform infrared (FTIR) spectrophotometer (Agilent Technology, Oxfordshire, UK) in attenuated total reflectance (ATR) mode. Spectra were collected in the 500–4000 cm^−1^ spectral range with a resolution of 4 cm^−1^ and an average of 8 scans.

#### 2.2.4. In Vitro Release Studies

The TA release from WPI hydrogels was measured using a spectrophotometer (Multi-Mode Reader Synergy H1, BioTek, Winooski, VT, USA) at 48 h after incubation. A dried hydrogel sample was weighed accurately and then incubated in PBS at room temperature for up to 48 h. At the indicated time, a few drops of 0.5 N iron(III) chloride were added to the selected aliquot, and the optical density of the solutions was measured at 586 nm (Figures S1 and S2) [24]. The tests were conducted on six independent replicates.

#### 2.2.5. Cell Viability Test

Cells were seeded in 96-well plates at the density described in the individual experiments. The following day, the excised hydrogel discs (diameter 3 mm) were added to triplicate wells. Fresh medium was added to each of 96 wells. Subsequently, the cells were incubated (Innova CO-170, New Brunswick Scientific, Enfield, CT, USA) at 37 °C for 48 h, together with the added materials. In the last step, 10 μL of AlamarBlue dye was added to each well and the intensity was measured using a spectrophotometer (Multi-Mode Reader Synergy H1). The experiment showed the capability of metabolically active cells to convert the AlamarBlue reagent into a fluorescent and colorimetric indicator. [25].

A commercially available laryngeal cancer cell line, Hep-2 (ATCC, CCL-23) was kindly provided by the center “Symbiosis” IBPPM RAS (Saratov, Russia).

#### 2.2.6. Statistical Analysis

The statistical data on the WPI-TA hydrogels’ swelling under conditions with different pH, both with and without TA, the TA release and the cytotoxic activity of the hydrogels were calculated using Microsoft Excel. Means and standard deviations were obtained from 3–6 independent experiments.

The data on the kinetics of swelling of hydrogels loaded with TA incubated in PBS at different pH values were plotted as “mean ± standard error” (*n* = 6). The viability of Hep2 cells incubated for 24 and 48 h with hydrogels containing different TA/WPI ratio was presented as “mean ± standard error” (*n* = 4). Differences between treatments were analyzed using two-way analysis of variance (ANOVA, Microsoft Excel 2016). [26] Calculations were carried out using Microsoft Excel software. Values of *p* ≤ 0.05 were considered significant (Tables S1–S4).

## 3. Results and Discussion

### 3.1. Preparation and Characterization of WPI Hydrogels Containing TAs

WPI is a promising cross-linking component for the preparation of hydrogels containing various biologically active compounds. Previously, hydrogels based on various WPI concentrations were synthesized and their properties were studied. [6]. Two types of TAs (polygalloyl glucoses—ALSOK 02, polygalloyl quinic acids—ALSOK 04) were used for the fabrication of the WPI hydrogels. The main differences in these preparations are varying amounts of hydroxyl groups and chemical structure. Based on the literature data, TA concentrations in WPI hydrogels were selected and hydrogels with differing TAs contents were synthesized 1.5; 3.0; 6.0 and 12.0 mg per mL, which corresponds to the TA/WPI ratios were 0.0375/0.075/0.15/0.30 in the hydrogels. [27,28]. Hydrogels were obtained by heating the solution to 90 °C for 30 min. Such a short exposure to high temperatures does not lead to pathological changes in the TA structure [29].

The gelification process of WPI-TAs solutions was carried out at pH 7 in deionized water. It is assumed that the incorporation of an additional small TAs amount into the WPI hydrogel structure (maximum TA/WPI ratio of 0.30) does not affect the hydrogel pI, since WPI is the prevailing constituent of hydrogels. According to previously published studies [30] the pI of hydrogels obtained at a pH above the native protein pI (pI 5.2) shifts to a more acidic range (pI 4.1) due to the electrostatic repulsion of negatively charged groups of glutamic and aspartic acids and corresponding deprotonation of lysine amino acid residues.

**Figure 1:**
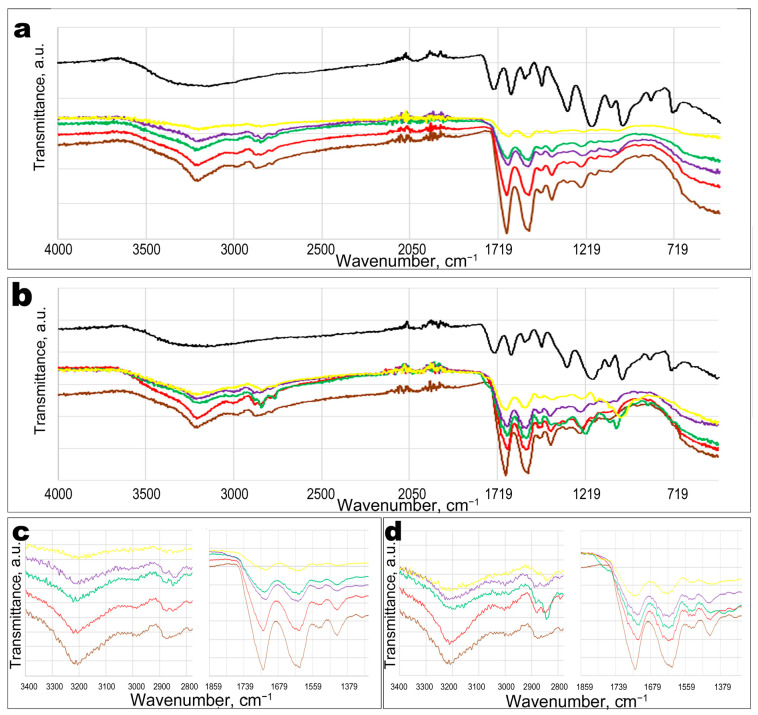
FTIR spectra of whey protein isolate (WPI) hydrogels with ALSOK 02 (**a**) and ALSOK 04 (**b**). Tannic acid (TA)/WPI ratio: 0.0 (burgundy line); 0.0375 (red line); 0.075 (green line); 0.15 (purple line); 0.30 (yellow line), TA (black line). The main diagnostic bands are magnified for FTIR spectra of WPI-ALSOK 02 (**c**) and WPI-ALSOK 04 (**d**) hydrogels. The dotted lines (**c**,**d**) indicate the main vibration signals (amide A, amide I, amide II) of the control WPI hydrogel without TAs (burgundy lines).

To understand the functional properties of WPI-TA hydrogels, it is necessary to determine their structure and identify the binding nature of the protein and polyphenols. FTIR measurements are a sensitive tool for detecting conformational changes in the secondary structure of a protein [31]. In the present study FTIR-spectra of WPI-TAs hydrogels were measured from a solid dried condition to exclude pronounced stretching vibrations of water molecules in the 3673–2942 cm^−1^ range and a deformation band of water in the 1644 cm^−1^ region. Figure 1 shows the FTIR spectra of unmodified WPI hydrogel and hydrogels with various TA concentrations. In the spectrum of the unmodified WPI hydrogel (burgundy line), we observed three strong bands at 3208, 1673, and 1545 cm^−1^, which correspond to vibrations for amide A, amide I, and amide II, respectively [32]. In the vibrational spectrum region of amide I, stretching vibrations of the COO^-^ of the Asn and Gln side residues and NH_3_^+^ deformation vibrations of amino acids containing additional NH_2_-groups in the side chain (Asp, Glu, Lys, and Arg) are manifested. This overlap of the amino acid residues absorption bands with the Amide I absorption band makes it very sensitive to the intermolecular H-bonds manifestation. A signal change of the amide I absorption band makes it possible to determine the conformational protein change.

FTIR spectra of hydrogels with different TA contents showed similar bands to that of the WPI hydrogel control spectrum. It indicates that new covalent bonds were not created. A similar result was reported by Ferraro, et al. (2015), who studied the nature of the interaction between rosmarinic acid (natural polyphenol) and milk whey proteins through non-covalent bonds in detail [33].

The spectral lines of hydrogels with TAs revealed broadening of the vibrational signal at 3208 cm^−1^, which indicates the formation of intermolecular H-bonds (Figure 1c,d). For hydrogels containing polygalloyl glucose (ALSOK 02) the broadening of the symmetric vibration signal of -NH and -OH groups into Amide A is more pronounced than for hydrogels with the same content of the polygalloyl quinine acid (ALSOK 04). H-bonds are the main binding force of WPI and hydrophilic substances [34]. Vibrational signals of Amide I and Amide II are considered the basis of the WPI signal and confirm the presence of whey proteins. A change in the secondary structure of the protein is usually explained by broadening of Amide I and a shift of Amide II. When more ALSOK 02 is added into hydrogels, the peaks of Amide I bending vibrations are shifted by 7 cm^−1^ (from 1545 cm^−1^ to 1538 cm^−1^) towards a lower wavenumber (Figure 1c). This indicates a change in the nature of the side amino group vibrations of Asp, Glu, Lys, and Arg due to the formation of intermolecular H-bonds with the polyphenols. The same phenomenon occurred for Amide II; the maximum shift was observed from 1673 cm^−1^ to 1657 cm^−1^ for a hydrogel with ALSOK 02/WPI ratio 0.30 (Figure 1b,d). For hydrogels containing ALSOK 04, the shifts of stretching vibrations of Amide I and Amide II groups were more significant than for hydrogels with ALSOK 02, perhaps due to the contribution of closely spaced signals of stretching vibrations of carboxyl groups and stretching of the C=C aromatic bonds of uncrosslinked ALSOK 04. The maximum shift was up to 25 cm^−1^ and was observed also for hydrogels with ALSOK 04/WPI ratio 0.30 (Figure 1d). The shift of Amide I and Amide II indicates the presence of an electrostatic interaction between WPI and TA, and not chemical reactions [31]. For the WPI-ALSOK 02 complex, the formation of intermolecular H-bonds is more characteristic than for the WPI-ALSOK 04, which directly depends on the chemical structure of TAs and their ability to ionize in water. Thus, a hydrolysable polygalloyl glucose (ALSOK 02) with a large number of hydroxyl groups interacts better with protein than polygalloyl quinic acid (ALSOK 04). Thus, in all cases, non-specific binding between polyphenols and WPI is confirmed, without additional covalent bond formation during the hydrogels’ preparation.

### 3.2. Swelling Kinetics of WPI Hydrogels

The swelling characteristics play an important role in the absorption of body fluids and the transfer of nutrients and cellular metabolites. One of the main strategies for releasing captured drugs is controlled hydrogel swelling. It is known that an osmotic pressure is also defined as the measure of the tendency of a solution to take in pure solvent by osmosis. Under an action of a solvent diffusion and hydrogel network osmotic pressure, an increase of the pore size is observed that results in mixing between the solvent and the WPI segments and, as a consequence, swelling of hydrogels [35]. The swelling degree of hydrogels depends on the stretching of the polymer chains, which exert a pressure inside the hydrogel through their elasticity.

**Figure 2:**
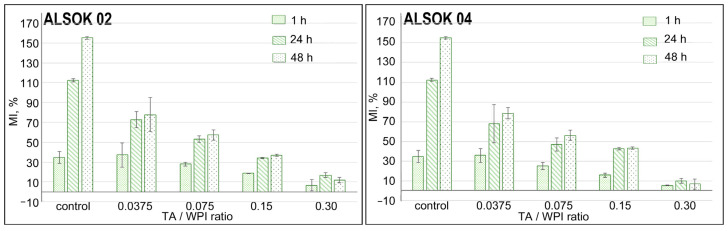
Mass increase (MI) of WPI hydrogels with TAs (ALSOK 02 (**left**), ALSOK 04 (**right**)) in PBS (pH 7). WPI hydrogel (control); TA/WPI ratios are 0.0375/0.075/0.15/0.30. *p* < 0.05 compared with the control groups (within each pH-dependence swelling group).

A swelling test was performed for WPI hydrogels containing different amounts of TAs and a control hydrogel without TAs in PBS solution (pH 7) for six repetitions within 48 h. The swelling degree of hydrogels depends on the hydrogel composition and the surrounding aqueous medium, as well as the degree of protein–protein, protein–water or protein–polyphenol interactions. [7] The increase of the mass increasing (MI) was observed for all hydrogels at the first 1 h of the swelling experiment (Figure 2). It indicates that all hydrogels absorbed and retained a certain amount of water in their structure. According to two-way analysis of variance (ANOVA), the swelling data of WPI-TA hydrogels are statistically significantly different (*p* < 0.05) between hydrogels with different TA/WPI ratio compared to the control hydrogels without TA. (Table S1).

As shown in Figure 2, the presence of TAs which are bound to WPI proteins by non-covalent electrostatic interaction in the hydrogel structure significantly reduces its swelling ability. The inability of TAs to absorb water reasons for the decrease in the MI of hydrogels thereby preventing swelling. Thus, a high polyphenol content in hydrogels can inhibit the penetration of various proteins and, therefore, it is believed that bioactive drugs will be protected from premature degradation due to the hindrance of enzyme diffusion into pores in the hydrogels. Also, the correlation between the swelling ratio of the hydrogel and the TA concentration will allow hindrance of drug diffusion into the body and, as a consequence, slow the kinetics of drug release [36]. The highest MI was observed for hydrogels with the lowest TAs/WPI ratio—0.0375.

In general, for hydrogels containing TAs with a high content of hydroxyl groups (ALSOK 02), the MI is higher than for hydrogels with the same concentration of polygalloylquinic acids (ALSOK 04). This is primarily due to the chemical structure of the added compounds. Addition of greater numbers of the hydroxyl groups to the hydrogel network allows an increase in the number of formed intermolecular H-bonds. As a rule, such bonds are labile and are easily stretched and broken by exposure to external stimuli. The osmotic pressure generated during the swelling process can be responsible for such spatial changes in the hydrogel networks.

An increase of the TA concentration in the WPI hydrogels reduces and limits the mobility of the hydrogel network, which leads to resistance to diffusion and water uptake. [37] So the smallest MI is observed for the hydrogels containing the maximum amount of ALSOK 02 and ALSOK 04. For hydrogels with a maximum TA content (TA/WPI ratio 0.30), complete swelling by water is observed 24 h after the incubation start. After 48 h, the MI decrease is observed (Figure 2) due to the subsequent reduction in the hydrogel mass, provoked, probably, by TA release from the hydrogel networks.

### 3.3. pH-Dependent Swelling Behaviors and TA Release from WPI Biohydrogels

**Figure 3:**
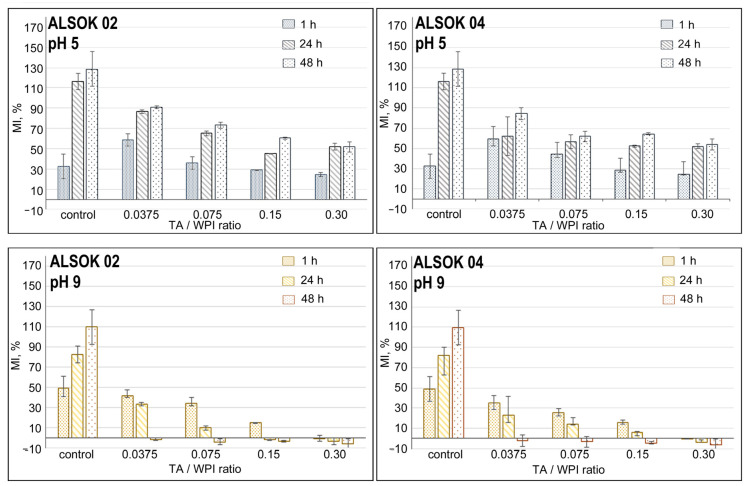
Mass increase (MI) of WPI hydrogels with TAs (ALSOK 02 (**left, up**) ALSOK 04 (**right, up**)) in PBS (pH 5). Mass increase (MI) of WPI hydrogels with TAs (ALSOK 02 (**left, down**) ALSOK 04 (**right, down**)) in PBS (pH 9). *p* < 0.05 compared with the control groups (within each pH-dependence swelling group).

We also focused on studying the pH dependence of hydrogel swelling. The prepared hydrogel compositions were immersed in acidic (pH 5, Figure 3 above) and basic (pH 9, Figure 3 below) phosphate buffered saline (PBS), incubated for 48 h at room temperature.

For 48 h after a storage, the solutions became more opaque in the basic state (pH 9), but transparent at acidic medium (pH 5). This is due to TA hydrolysis and the subsequent oxidation by decarboxylation of the hydrolysis products in the presence of base. Usually, hydrogels formed from amphoteric polyelectrolytes (for example, WPI) have a small MI at a pH equal to their isoelectric point (pI of native ß-lactoglobulin is 5.1) [38]. The presence of a high TAs content affects the diffusion of ions, reducing the elasticity of the hydrogel network. Such a low ability of hydrogels to take up water is associated with less interaction or absence of WPI hydrophilic sites with water due to the formation of numerous bonds between the protein and TAs. Due to this, the formation of denser and more rigid structures occurs, which leads to a decrease in the flexibility of protein chains. In PBS solutions, the swelling capacity of hydrogels is lower compared to the values in distilled water. This can be explained by the uneven distribution of ions in the hydrogel network and solution. This causes a decrease in the equilibrium water absorption of the hydrogel and a swelling decrease over time.

**Figure 4:**
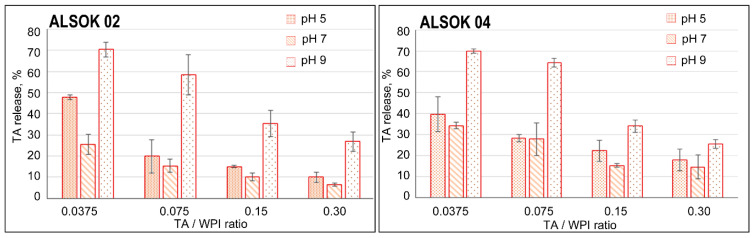
Histograms of the released TA amount (ALSOK 02 (**left**) ALSOK 04 (**right**)) from the WPI hydrogel at 48 h after incubation. Error bars show standard errors.

It is interesting to note the behavior of hydrogels in the basic medium (Figure 4 left, down; right, down). The MI value for hydrogels at pH 9 is higher than at pH 2 during the first hour of the experiment. So the higher the pH, the more surface charges, the higher the electrostatic repulsive force, and higher MI value [30,39]. For the control WPI sample that does not contain TAs, the MI value continues to grow throughout the duration of the experiment. However, the presence of TA in the hydrogel results in lower MI values. According to two-way analysis of variance (ANOVA), statistically significant differences (*p* < 0.05) in the swelling data of hydrogels are observed between hydrogels with different TA/WPI ratio compared to the control hydrogels without TA (Tables S2 and S3). A decrease of MI values is observed with increasing TA concentration in the hydrogels. Due to the hydrolysis of TAs under basic conditions and partial deprotonization, the destruction of intermolecular H-bonds is possible and, as a consequence, the release of TA hydrolysis products from hydrogels with subsequent weight loss. We do not exclude the possibility that WPI material may be diffusing out of the hydrogels too. Future work will investigate the possible simultaneous release of hydrogel material.

Targeted drug release from hydrogels in combination with a controlled release rate is a desirable property of pH-sensitive hydrogels. To confirm its hypothesis, the TA release from hydrogels was studied at different pH. Figure 4 shows the TA release profiles from hydrogels 48 h after their incubation in PBS solution at pH 5, 7, and 9, respectively.

According to Figure 4, TA release was the smallest when the samples were immersed in a neutral medium (pH 7). It is believed that the strongest ionic interaction between polyphenols and protein occurs in the solution at pH was close to the isoelectric point of native whey proteins (pI 5.1) [40], which leads to the formation of a denser hydrogels.

The highest TAs release 48 h after incubation is observed for hydrogels in the basic medium (pH 9), which is consistent with the swelling test data. An increase in pH will lead to deprotonation of WPI and TAs. As a result, a large TA release percentage is observed, which is associated with a violation of intermolecular H-bonds [41]. For hydrogels containing a small TA weight (TAs/WPI ratio—0.0375) the TA release percentage reaches high values, up to 80%. However, for WPI hydrogels with the highest TA content (TAs/WPI ratio—0.30), only 40% of the TA initially present is released from the hydrogel network. It leads to the formation of a denser hydrogel. We do not exclude the possibility that WPI material may be diffusing out of the hydrogels. Our future work will investigate the possibility of simultaneous release of hydrogel material. This aspect is important for the development of hydrogel scaffold with controlled release of drugs and nutrients, as well as the case of wound healing, absorption of wound exudates.

In an acidic medium (pH 5), a high TAs release value is observed, which is also associated with protein dissociation and protonation. This may be a positive sign for effective cancer therapy, since the local and endosomal pH is significantly lower than that of normal tissue [42].

Thus, the pH-dependent drug release from hydrogels allows hydrogels to be used locally, as anticancer scaffolds for the treatment or palliative treatment of serious gastrointestinal malignancies where pH values range from acidic (in the stomach) to basic (in the intestine).

### 3.4. Anticancer Activity of WPI Hydrogels Containing TA

Cytotoxicity of WPI hydrogels was estimated on the laryngeal cancer cell line (Hep 2) using the Alamar Blue assay, which measures the metabolic activity of cells.

**Figure 5:**
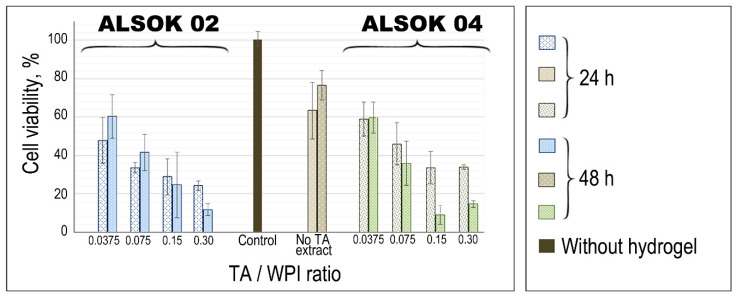
Results of cytotoxicity tests of WPI hydrogels without and with addition of TAs (ALSOK 02 and ALSOK 04) on the Hep 2 cell line. The Hep 2 cells were cultured in the presence of WPI hydrogels containing TAs (blue—ALSOK 02, green—ALSOK 04), hydrogels without TAs (brown). Cell culture without adding WPI hydrogels (black) was the control throughout the experiment. *p* < 0.05 compared with the control groups without adding TA.

The cultivation of Hep 2 cells during 48 h in the presence of WPI hydrogel discs without and with the addition of TAs (ALSOK 02, ALSOK 04) showed that samples without TAs exerted an inconsiderable cytotoxic effect on the cell line whereas hydrogels contained TAs caused a significant inhibition of metabolic processes (Figure 5).

Hydrogels with TA/WPI ratio 0.0375 produced a similar effect in comparison to pure hydrogel samples. The increase of TAs concentration led to more significant cytotoxic effects, correspondingly. Samples with maximum TA/WPI ratio 0.3 after 24 h incubation exhibited to 50% inhibition of metabolic processes whereas after 48 h this value increased to 80%. Previously, the ability of polyphenol derivatives to induce apoptosis and cell cycle termination was shown for cancer cell lines in vitro [43,44]. However, the cytotoxic effect of hydrogels with ALSOK 02 was higher than for the sample containing ALSOK 04 (Figure 5). Significant differences in cell viability between WPI hydrogels with different TA/WPI ratio were observed (*p* < 0.05) compared with the control groups without adding TA for each one of TA types (Table S4). In previous work on mineralized gellan gum hydrogels containing ALSOK 02 and ALSOK 04, greater cytotoxicity towards osteosarcoma-derived Saos-2 cells was observed after 2 h [22]. Thus, the use of WPI hydrogels containing TAs at 3 mg per mL (TA/WPI ratio 0.075) concentration is the most promising for provision of a prolonged anti-cancer effect.

## 4. Conclusions and Outlook

WPI hydrogels containing two types of TA have been produced, which withstand autoclaving. The greatest influence on the swelling change is exerted by the amount of TAs contained in the WPI hydrogels. An increase of the TA/WPI ratio in the hydrogels to 0.30 (for ALSOK 02 and ALSOK 04 both) leads to a significant decrease in MI compared with the control hydrogel without TA in neutral conditions (pH 7). The pH lowering leads to a MI decrease and an increase in the amount of released TAs by 1.5–2 times compared with incubation at neutral pH (pH 7) for all WPI hydrogels with and without TAs. The maximum TAs release was observed for hydrogels with the TA/WPI ratio 0.0375 (for ALSOK 02 and ALSOK 04 both) in alkaline pH (pH 9) and amounted to almost 80% 48 h after the incubation start. According to the swelling data, at this time point, the hydrogels begin to destruct, since their MI have negative values at 48 h. Future work will investigate the possible simultaneous release of hydrogel material. Also, measurements of the pH and zeta potential of the hydrogel dependence on pH gelification will be investigated in our future work. All obtained hydrogels containing TAs have cytotoxic properties against the human laryngeal cell carcinoma (Hep-2) Hep-2 cell line. An increase in the concentration of TAs in hydrogels leads to an increase in the cytotoxic effect. Thus, a WPI hydrogels can be used as anti-cancer scaffolds with a prolonged release profile of TAs.

## Data Availability

The data that support the findings of this study are contained within the article.

## Supplementary Materials

The following are available online at https://www.mdpi.com/1996-1944/14/4/749/s1, Figure S1: Linearity using ALSOK 02 demonstrating the line equation and the linear correlation coefficient (R2), Figure S2: Linearity using ALSOK 04 demonstrating the line equation and the linear correlation coefficient (R2), Table S1: *p*-Values for two-way analysis of variance (ANOVA) statistics for the WPI-TA hydrogel`s swelling data (TA/WPI ratio–0.0375/0.075/0.15/0.3) at pH 7 compared to the swelling data of the WPI hydrogel without TA, Table S2: *p*-Values for two-way analysis of variance (ANOVA) statistics for the WPI-TA hydrogel’s swelling data (TA/WPI ratio–0.0375/0.075/0.15/0.3) at pH 5 compared to the swelling data of the WPI hydrogel without TA, Table S3: *p*-Values for two-way analysis of variance (ANOVA) statistics for the WPI-TA hydrogel’s swelling data (TA/WPI ratio–0.0375/0.075/0.15/0.3) at pH 9 compared to the swelling data of the WPI hydrogel without TA, Table S4: *p*-Values for two-way analysis of variance (ANOVA) statistics for the cell viability data of WPI-TA hydrogels (TA/WPI ratio–0.0375/0.075/0.15/0.3) compared to the WPI hydrogel without TA.

## Abbreviations

FTIR	Fourier transform infrared
H-bond	Hydrogen bond
MI	Mass increase
PBS	Phosphate buffered saline
TA	Tannic acid
WPI	Whey protein isolate

## References

[1] Gupta D., Kocot M., Tryba A.M., Serafim A., Stancu I.C., Jaegermann Z., Pamuła E., Reilly G.C., Douglas T.E.L. (2020). Novel naturally derived whey protein isolate and aragonite biocomposite hydrogels have potential for bone regeneration. Mater. Des..

[2] Norris K., Kocot M., Tryba A.M., Chai F., Talari A., Ashton L., Parakhonskiy B.V., Samal S.K., Blanchemain N., Pamuła E. (2020). Marine-Inspired Enzymatic Mineralization of Dairy-Derived Whey Protein Isolate (WPI) Hydrogels for Bone Tissue Regeneration. Mar. Drugs.

[3] Dziadek M., Douglas T.E.L., Dziadek K., Zagrajczuk B., Serafim A., Stancu I.C., Cholewa-Kowalska K. (2020). Novel whey protein isolate-based highly porous scaffolds modified with therapeutic ion-releasing bioactive glasses. Mater. Lett..

[4] Dziadek M., Kudlackova R., Zima A., Slosarczyk A., Ziabka M., Jelen P., Shkarina S., Cecilia A., Zuber M., Baumbach T. (2019). Novel multicomponent organic–inorganic WPI/gelatin/CaP hydrogel composites for bone tissue engineering. J. Biomed. Mater. Res. Part A.

[5] Lorenzen P.C., Schrader K. (2006). A comparative study of the gelation properties of whey protein concentrate and whey protein isolate. Lait.

[6] Douglas T.E.L., Vandrovcová M., Kročilová N., Keppler J.K., Zárubová J., Skirtach A.G., Bačáková L. (2018). Application of whey protein isolate in bone regeneration: Effects on growth and osteogenic differentiation of bone-forming cells. J. Dairy Sci..

[7] Ozel B., Cikrikci S., Aydin O., Oztop M.H. (2017). Polysaccharide blended whey protein isolate-(WPI) hydrogels: A physicochemical and controlled release study. Food Hydrocoll..

[8] Andoyo R., Lestari V.D., Mardawati E., Nurhadi B. (2018). Fractal Dimension Analysis of Texture Formation of Whey Protein-Based Foods. Int. J. Food Sci..

[9] Chatterjee S., Chi-leung HUI P. (2019). Review of Stimuli-Responsive Polymers in Drug Delivery and Textile Application. Molecules.

[10] Jourdes M., Pouységu L., Deffieux D., Teissedre P.L., Quideau S. (2013). Hydrolyzable tannins: Gallotannins and ellagitannins. Natural Products: Phytochemistry, Botany and Metabolism of Alkaloids, Phenolics and Terpenes.

[11] Ky I., Le Floch A., Zeng L., Pechamat L., Jourdes M., Teissedre P.L. (2015). Tannins. Encyclopedia of Food and Health.

[12] Zaborniak I., Chmielarz P., Wolski K., Grzes´ G., Isse A.A., Gennaro A., Zapotoczny S., Sobkowiak A. (2019). Tannic Acid-Inspired Star-Like Macromolecules via Temporally Controlled Multi-Step Potential Electrolysis. Macromol. Chem. Phys..

[13] Isenburg J.C., Karamchandani N.V., Simionescu D.T., Vyavahare N.R. (2006). Structural requirements for stabilization of vascular elastin by polyphenolic tannins. Biomaterials.

[14] Pranantyo D., Xu L.Q., Neoh K.G., Kang E.T., Ng Y.X., Teo S.L.M. (2015). Tea Stains-Inspired Initiator Primer for Surface Grafting of Antifouling and Antimicrobial Polymer Brush Coatings. Biomacromolecules.

[15] Chai Y., Lee H.J., Shaik A.A., Nkhata K., Xing C., Zhang J., Jeong S.J., Kim S.H., Lü J. (2010). Penta-O-galloyl-β-D-glucose induces G1arrest and DNA replicative S-phase arrest independently of P21 cyclin-dependent kinase inhibitor 1A, P27 cyclin-dependent kinase inhibitor 1B and P53 in human breast cancer cells and is orally active against triple-negative xenograft growth. Breast Cancer Res..

[16] He J., Dong Y., Liu X., Wan Y., Gu T., Zhou X., Liu M. (2019). Comparison of Chemical Compositions, Antioxidant, and Anti-Photoaging Activities of Paeonia suffruticosa Flowers at Different Flowering Stages. Antioxidants.

[17] Mendonca P., Taka E., Soliman K.F.A. (2019). Proteomic analysis of the effect of the polyphenol pentagalloyl glucose on proteins involved in neurodegenerative diseases in activated BV-2 microglial cells. Mol. Med. Rep..

[18] Bridgeman C.J., Nguyen T.-U., Kishore V. (2018). Anticancer efficacy of tannic acid is dependent on the stiffness of the underlying matrix. J. Biomater. Sci. Polym. Ed..

[19] Boyle W.S., Chen W., Rodriguez A., Linn S., Tolar J., Lozano K., Reineke T.M. (2019). Ternary Composite Nanofibers Containing Chondroitin Sulfate Scavenge Inflammatory Chemokines from Solution and Prohibit Squamous Cell Carcinoma Migration. ACS Appl. Bio. Mater..

[20] Ge W., Cao S., Shen F., Wang Y., Ren J., Wang X. (2019). Rapid self-healing, stretchable, moldable, antioxidant and antibacterial tannic acid-cellulose nanofibril composite hydrogels. Carbohydr. Polym..

[21] Seczyk L., Swieca M., Kapusta I., Gawlik-Dziki U. (2019). Protein–phenolic interactions as a factor affecting the physicochemical properties of white bean proteins. Molecules.

[22] Douglas T.E.L., Keppler J.K., Vandrovcová M., Plencner M., Beranová J., Feuereisen M., Parakhonskiy B.V., Svenskaya Y., Atkin V., Ivanova A. (2020). Enhancement of Biomimetic Enzymatic Mineralization of Gellan Gum Polysaccharide Hydrogels by Plant-Derived Gallotannins. Int. J. Mol. Sci..

[23] Carson M., Keppler J.K., Brackman G., Dawood D., Vandrovcova M., Fawzy El-Sayed K., Coenye T., Schwarz K., Clarke S.A., Skirtach A.G. (2018). Whey Protein Complexes with Green Tea Polyphenols: Antimicrobial, Osteoblast-Stimulatory, and Antioxidant Activities. Cells Tissues Organs.

[24] Bray H.G., Thorpe W.V. (1954). Analysis of phenolic compounds of interest in metabolism. Methods Biochem. Anal..

[25] Back S.A., Khan R., Gan X., Rosenberg P.A., Volpe J.J. (1999). A new Alamar Blue viability assay to rapidly quantify oligodendrocyte death. J. Neurosci. Methods.

[26] In J., Lee S. (2017). Statistical data presentation. Korean J. Anesthesiol..

[27] Ngobili T.A., Shah H., Park J.P., Kwist K.W., Inskeep B., Burg K.J.L., Booth B.W. (2015). Remodeling of tannic acid crosslinked collagen type I induces apoptosis in ER+ breast cancer cells. Anticancer Res..

[28] Karakurt S., Adali O. (2016). Tannic Acid Inhibits Proliferation, Migration, Invasion of Prostate Cancer and Modulates Drug Metabolizing and Antioxidant Enzymes. Anticancer. Agents Med. Chem..

[29] Wang C.-C., Chen H.-F., Wu J.-Y., Chen L.-G. (2019). Stability of Principal Hydrolysable Tannins from Trapa taiwanensis Hulls. Molecules.

[30] Betz M., Hörmansperger J., Fuchs T., Kulozik U. (2012). Swelling behaviour, charge and mesh size of thermal protein hydrogels as influenced by pH during gelation. Soft Matter..

[31] Jia Z., Zheng M., Tao F., Chen W., Huang G., Jiang J. (2016). Effect of covalent modification by (-)-epigallocatechin-3-gallate on physicochemical and functional properties of whey protein isolate. LWT Food Sci. Technol..

[32] Jackson M., Mantsch H.H. (1995). The use and misuse of FTIR spectroscopy in the determination of protein structure. Crit. Rev. Biochem. Mol. Biol..

[33] Ferraro V., Madureira A.R., Sarmento B., Gomes A., Pintado M.E. (2015). Study of the interactions between rosmarinic acid and bovine milk whey protein α-Lactalbumin, β-Lactoglobulin and Lactoferrin. Food Res. Int..

[34] Wang C., Zhou X., Wang H., Sun X., Guo M. (2019). Interactions between β-Lactoglobulin and 3,3′-Diindolylmethane in Model System. Molecules.

[35] Barros W. (2019). Solvent self-diffusion dependence on the swelling degree of a hydrogel. Phys. Rev. E.

[36] Kang G.D., Cheon S.H., Song S.C. (2006). Controlled release of doxorubicin from thermosensitive poly(organophosphazene) hydrogels. Int. J. Pharm..

[37] Barros J., Ferraz M.P., Azeredo J., Fernandes M.H., Gomes P.S., Monteiro F.J. (2019). Alginate-nanohydroxyapatite hydrogel system: Optimizing the formulation for enhanced bone regeneration. Mater. Sci. Eng. C.

[38] Ozdal T., Capanoglu E., Altay F. (2013). A review on protein-phenolic interactions and associated changes. Food Res. Int..

[39] Le Bourvellec C., Renard C.M.G.C. (2012). Interactions between polyphenols and macromolecules: Quantification methods and mechanisms. Crit. Rev. Food Sci. Nutr..

[40] Gunasekaran S., Ko S., Xiao L. (2007). Use of whey proteins for encapsulation and controlled delivery applications. J. Food Eng..

[41] Jiang J., Zhang Z., Zhao J., Liu Y. (2018). The effect of non-covalent interaction of chlorogenic acid with whey protein and casein on physicochemical and radical-scavenging activity of in vitro protein digests. Food Chem..

[42] Damaghi M., Wojtkowiak J.W., Gillies R.J. (2013). pH sensing and regulation in cancer. Front. Physiol..

[43] Kwon H.Y., Kim J.H., Kim B., Srivastava S.K., Kim S.H. (2018). Regulation of SIRT1/AMPK axis is critically involved in gallotannin-induced senescence and impaired autophagy leading to cell death in hepatocellular carcinoma cells. Arch. Toxicol..

[44] Park E., Kwon H.Y., Jung J.H., Jung D.B., Jeong A., Cheon J., Kim B., Kim S.H. (2015). Inhibition of Myeloid Cell Leukemia 1 and Activation of Caspases Are Critically Involved in Gallotannin-induced Apoptosis in Prostate Cancer Cells. Phyther. Res..

