# Supplementary material for: pH-Sensitive Dairy-Derived Hydrogels with a Prolonged Drug Release Profile for Cancer Treatment
Source: Materials (Basel). 2021 Feb 5;14(4):749. doi: 10.3390/ma14040749 (PMC7915325; doi:10.3390/ma14040749)
Supplement: Supplementary file 1 [file materials-14-00749-s001.pdf]

materials

Supplementary Material

pH-Sensitive Dairy-Derived Hydrogels with a Prolonged Drug Release Profile for Cancer Treatment

Oksana A. Mayorova [1,*], Ben C.N. Jolly [2], Roman A. Verkhovskii [1], Valentina O. Plastun [1], Olga A. Sindeeva [1,3] and Timothy E.L. Douglas [2,4,*]

[1] Institute of Nanostructures and Biosystems, Saratov State University, 83 Astrakhanskaya st., 410012 Saratov, Russia; r.a.verhovskiy@mail.ru (R.A.V.); voplastun@gmail.com (V.O.P.); o.sindeeva@skoltech.ru (O.A.S.)
[2] Engineering Department, Lancaster University, Gillow Av., Lancaster LA1 4YW, UK; bcnjolly001@gmail.com
[3] Skolkovo Institute of Science and Technology, Skolkovo Innovation Center, Building 3, 143026 Moscow, Russia
[4] Materials Science Institute (MSI), Lancaster University, Gillow Av., Lancaster LA1 4YW, UK
* Correspondence: Beloousova011@yandex.ru (O.A.M.); t.douglas@lancaster.ac.uk (T.E.L.D.)

Citation: Mayorova, O.A.; Jolly, B.C.N.; Verkhovskii, R.A.; Plastun, V.O.; Sindeeva, O.A.; Douglas, T.E.L. pH-Sensitive Dairy-Derived Hydrogels with a Prolonged Drug Release Profile for Cancer Treatment. *Materials* **2021**, *14*, 749. https://doi.org/10.3390/ma14040749

Received: 16 December 2020
Accepted: 29 January 2021
Published: 5 February 2021

Publisher's Note: MDPI stays neutral with regard to jurisdictional claims in published maps and institutional affiliations.

Copyright: © 2021 by the authors. Submitted for possible open access publication under the terms and conditions of the Creative Commons Attribution (CC BY) license (http://creativecommons.org/licenses/by/4.0/).

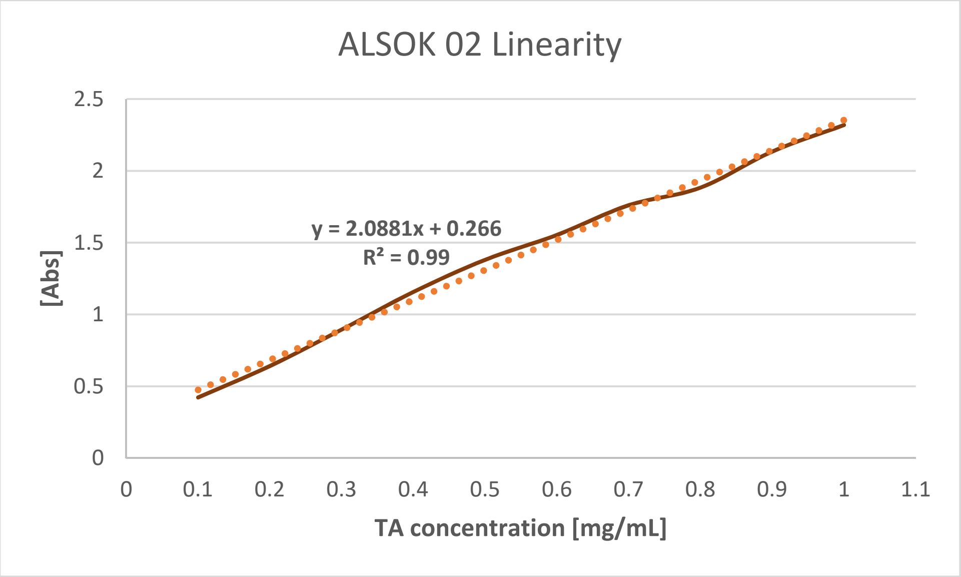

Figure S1. Linearity using ALSOK 02 demonstrating the line equation and the linear correlation coefficient (R^2).

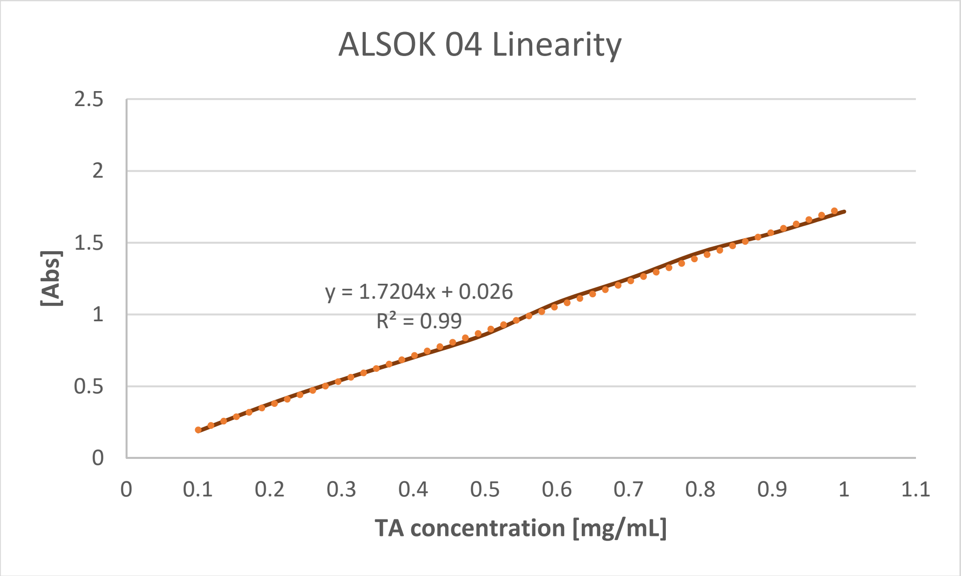

Figure S2. Linearity using ALSOK 04 demonstrating the line equation and the linear correlation coefficient (R^2).

	ALSOK 02				ALSOK 04			
	0.0375	0.075	0.15	0.3	0.0375	0.075	0.15	0.3
1 h	0.0160	0.0101	0.0035	0.0298	0.0108	0.0122	0.0015	0.0207
24 h	0.0014	0.0049	0.0088	0.0073	0.0252	0.0139	0.0248	0.0312
48 h	0.0306	0.0402	0.0096	0.0085	0.0074	0.0089	0.0067	0.0072

Table S1. P-Values for two-way analysis of variance (ANOVA) statistics for the WPI-TA hydrogel swelling data (TA/WPI ratio—0.0375/0.075/0.15/0.3) at pH 7 compared to the swelling data of the WPI hydrogel without TA.

	ALSOK 02				ALSOK 04			
	0.0375	0.075	0.15	0.3	0.0375	0.075	0.15	0.3
1 h	0.0439	0.0358	0.0347	0.0022	0.0173	0.0105	0.0255	0.0079
24 h	0.0197	0.0363	0.0039	0.0074	0.0384	0.0199	0.0131	0.0364
48 h	0.0246	0.0034	0.0137	0.0141	0.0059	0.0272	0.0186	0.0187

Table S2. P-Values for two-way analysis of variance (ANOVA) statistics for the WPI-TA hydrogel swelling data (TA/WPI ratio—0.0375/0.075/0.15/0.3) at pH 5 compared to the swelling data of the WPI hydrogel without TA.

	ALSOK 02				ALSOK 04			
	0.0375	0.075	0.15	0.3	0.0375	0.075	0.15	0.3
1 h	0.0115	0.0334	0.0015	0.0314	0.0047	0.0411	0.0093	0.0014
24 h	0.0313	0.0011	0.0278	0.0014	0.0379	0.0069	0.0410	0.0163
48 h	0.0071	0.0412	0.0056	0.0360	0.0115	0.0014	0.0018	0.0061

Table S3. P-Values for two-way analysis of variance (ANOVA) statistics for the WPI-TA hydrogel swelling data (TA/WPI ratio—0.0375/0.075/0.15/0.3) at pH 9 compared to the swelling data of the WPI hydrogel without TA.

	ALSOK 02				ALSOK 04			
	0.0375	0.075	0.15	0.3	0.0375	0.075	0.15	0.3
24 h	0.0293	0.0467	0.0302	0.0194	0.0399	0.0145	0.0495	0.0290
48 h	0.0118	0.0073	0.0037	0.0010	0.0487	0.0021	0.0020	0.0005

Table S4. P-Values for two-way analysis of variance (ANOVA) statistics for the cell viability data of WPI-TA hydrogels (TA/WPI ratio—0.0375/0.075/0.15/0.3) compared to the WPI hydrogel without TA.
